# CD8^+^ T-cell responses in HIV controllers: potential implications for novel HIV remission strategies

**DOI:** 10.1097/COH.0000000000000748

**Published:** 2022-07-01

**Authors:** Rachel L. Rutishauser, Lydie Trautmann

**Affiliations:** a Department of Medicine, University of California, San Francisco, San Francisco, California; b Gladstone-UCSF Institute for Genomic Immunology, San Francisco, CA, USA; c Vaccine and Gene Therapy Institute, Oregon Health & Science University, Beaverton, Oregon, USA

**Keywords:** CD8^+^ T cells, HIV controllers, HIV remission strategies

## Abstract

Immunological studies of spontaneous HIV and simian virus (SIV) controllers have identified virus-specific CD8^+^ T cells as a key immune mechanism of viral control. The purpose of this review is to consider how knowledge about the mechanisms that are associated with CD8^+^ T cell control of HIV/SIV in natural infection can be harnessed in HIV remission strategies.

**Recent findings:**

We discuss characteristics of CD8^+^ T-cell responses that may be critical for suppressing HIV replication in spontaneous controllers comprising HIV antigen recognition including specific human leukocyte antigen types, broadly cross-reactive T cell receptors and epitope targeting, enhanced expansion and antiviral functions, and localization of virus-specific T cells near sites of reservoir persistence. We also discuss the need to better understand the timing of CD8^+^ T-cell responses associated with viral control of HIV/SIV during acute infection and after treatment interruption as well as the mechanisms by which HIV/SIV-specific CD8^+^ T cells coordinate with other immune responses to achieve control.

**Summary:**

We propose implications as to how this knowledge from natural infection can be applied in the design and evaluation of CD8^+^ T-cell-based remission strategies and offer questions to consider as these strategies target distinct CD8^+^ T-cell-dependent mechanisms of viral control.

## INTRODUCTION: THE ROLE OF CD8^+^ T-CELL RESPONSES IN HIV CONTROLLERS

In HIV infection as in any other viral infection, disease progression and outcome are highly variable between individuals and a wide spectrum of viral control exists that is dependent on virologic features, route of transmission, host genetics, immune responses and environment. While the vast majority of people living with HIV are unable to suppress viral replication without antiretroviral therapy (ART), a small group of people – less than 1% of people living with HIV-1 and ∼15% of people living with HIV-2, with an increased proportion of women – can naturally keep the virus under control [1,2]. Over the years, these individuals exhibiting spontaneous control of HIV replication have been termed long-term nonprogressors, viremic controllers, or elite controllers based on different virologic and clinical criteria [3]. Elite controllers, whose viral loads in the plasma can remain undetectable for decades in the absence of ART, have a wide range of HIV reservoir sizes [4,5]. Recently, a rare sub-group of elite controllers termed ‘exceptional’ elite controllers have been found to harbor an extremely small HIV reservoir, thus achieving a state very close to a natural HIV remission [5,6^▪^]. In addition to the individuals who control HIV in the absence of ART, two small cohorts of people who initially required ART to control viremia and were later found to suppress HIV replication after stopping ART have been identified and called posttreatment controllers (PTCs; [7–9]), a phenotype that may be more frequent if ART is initiated early in the course of infection.

Evidence is strong that CD8^+^ T cells can play a critical role in mediating control of HIV in some controllers. From studies in humans with HIV and nonhuman primates (NHPs) with simian viruses (SIV, SHIV), potent CD8^+^ T-cell responses have been shown to associate with a lower viral load setpoint in both acute infection and after treatment interruption [10–14]. In elite controllers, as we will discuss, the role of HIV-specific CD8^+^ T-cell responses has been suggested by the association between viral control and specific enrichment of class I human or primate leukocyte antigen (HLA or Mamu) alleles and the development of potent HIV/SIV-specific CD8^+^ T-cell responses that are independent of the HLA/Mamu type [15–29]. More directly, depletion of CD8^+^ T cells has been shown to lead to viral rebound in NHPs that have either spontaneous elite control or viral suppression induced after treatment in early infection with broadly neutralizing antibodies [30^▪^,^31^–^33^]. In contrast, waning antibody titers and sero-reversion suggest a fading humoral response in the rare exceptional elite controllers but, to date, no data have been reported on the CD8^+^ T-cell responses in these people. PTCs have so far not demonstrated the HLA genetic characteristics seen in elite controllers and early data suggest that they may have a low magnitude HIV-specific CD8^+^ T-cell response, at least as measured by interferon gamma production after HIV peptide stimulation [8]. PTCs may have diverse mechanisms of control: some may have mechanisms attributed to natural killer (NK) cells for viral control but it is possible that, at least in some PTCs, CD8^+^ T cells play a role in viral suppression and that early treatment could also help to preserve CD8^+^ T-cell function [34,35].

**FIGURE 1:**
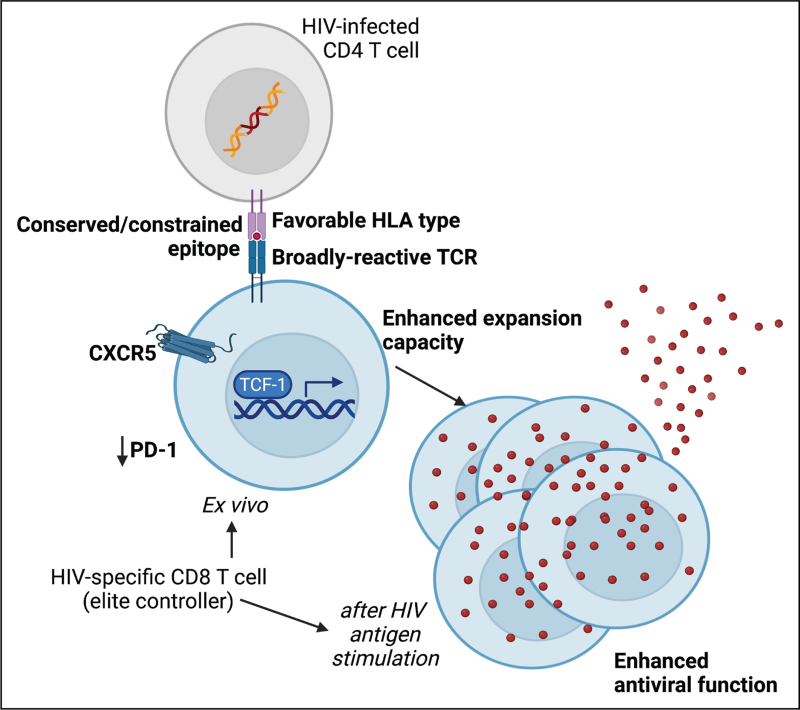
Key CD8^+^ T-cell features to target for HIV remission. HIV-specific CD8^+^ T cells from elite controllers are more likely to be restricted by specific HLA types, target epitopes derived from the relatively evolutionarily conserved/constrained regions of the HIV genome, and to have broadly-reactive T cell receptors (TCRs) capable of recognizing variant epitopes. Evaluated directly *ex vivo*, they occupy a T cell memory-like differentiation state with high TCF-1 expression and low levels of expression of coinhibitory receptors such as PD-1 and they are more likely to accumulate in B cell follicles in lymphoid tissue (due to expression of CXCR5). After stimulation with HIV antigens *in vitro*, they demonstrate enhanced expansion capacity and an ability to generate secondary effector cells that have enhanced antiviral function.

In this review, we first discuss features of CD8^+^ T cells that are associated with and may contribute to spontaneous control of infection in elite controllers (Fig. 1) and consider how each of these features might be leveraged to inform novel CD8^+^ T-cell-based HIV remission strategies. Next, we discuss the importance of studying how the timing of CD8^+^ T-cell responses and the coordination between CD8^+^ T cells and other immune responses relates to HIV/SIV control both in acute infection and after treatment interruption. Finally, we discuss how this information might be used to successfully apply knowledge from HIV controllers to the design of novel therapies and clinical trials to induce HIV remission. 

**Box 1:**
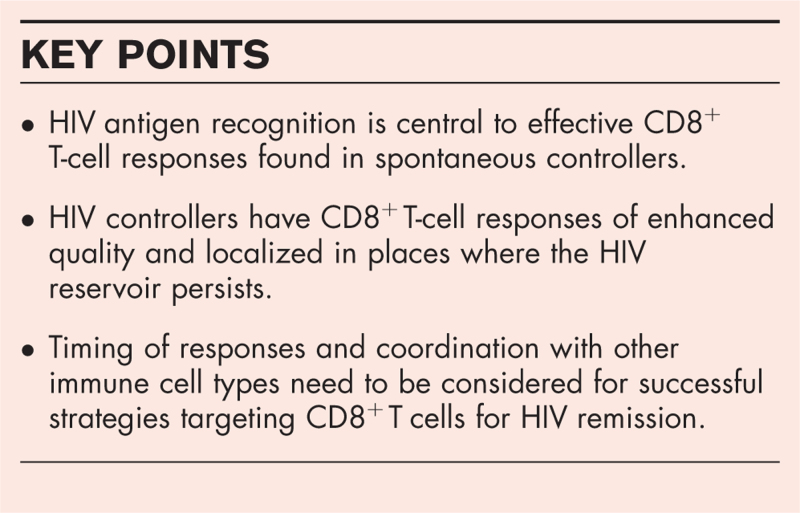
no caption available

### I. HIV antigen recognition by CD8^+^ T cells in elite controllers

Given the evidence that CD8^+^ T cells play an important role in viral control in HIV infection, there has been intense investigation over the years into the question of whether specific epitope targeting and/or features of T-cell receptor (TCR) recognition of viral peptides in natural infection favors viral control. This concept has been supported by three major lines of evidence: first, there is well documented association of elite controller status with protective class I HLA alleles; second, viral control has been associated with more cross-reactive ‘public’ TCRs (i.e., TCRs with CRD3 regions shared across different people); third, there is evidence that CD8^+^ T cells from spontaneous controllers are more likely to target highly mutationally constrained – or ‘networked’ – epitopes.

#### Human leukocyte antigen restriction

Across the human population, polymorphisms within the HLA locus provide one mechanism for genetically encoded inter-individual variation in epitope targeting. As reviewed elsewhere [15,25], independent studies have identified strong associations between specific class I *HLA* alleles and increased likelihood of elite control (e.g., *HLA-B*^*∗*^*57*, *HLA-B*^*∗*^*27*, *HLA-B*^*∗*^*52* and *HLA-B*^*∗*^*14*), although the presence of these alleles is neither necessary nor sufficient to predict controller status [17,19]. Spontaneous control of HIV has been associated with specific polymorphisms in the amino acids lining the HLA class I peptide-binding groove [19], which likely influences the specific viral peptides that are presented to CD8^+^ T cells in individuals with those polymorphisms. While the population diversity of classical class I HLA alleles is vast, CD8^+^ T cells can also be restricted by HLA-E, a nonclassical major histocompatibility complex (MHC) protein with only two alleles found in worldwide populations [36]. Strikingly, cytomegalovirus (CMV)-vectored SIV vaccines can elicit broad HLA-E-restricted SIV-specific CD8^+^ T cell responses that are associated with the prevention of an established chronic infection in ∼50% of animals upon viral challenge [37,38]. HLA-E-restricted responses have been detected in people with HIV but have not yet been associated with viral control [39^▪^,^40^]. Implications:

- HLA type should be characterized and accounted for in the immunogenicity and efficacy analysis of all HIV remission studies.
- T-cell-based remission strategies should aim to elicit responses across diverse HLA types.
- Could targeting of HLA-E-restricted HIV-specific CD8^+^ T cells offer a more universal approach to therapeutic vaccination for HIV?

#### ‘Broadly reactive’ T-cell receptors

In addition to favorable HLA alleles, people who spontaneously control HIV are more likely to have HIV-specific CD8^+^ T-cell responses consisting of TCRs with higher avidity and more cross-reactive public clonotypes [41–43]. *HLA* alleles associated with HIV control promote thymic selection of more TCR repertoires that have cross-reactivity for viral variants [44]. Additionally, within the same HLA-B^∗^27-restricted epitope response, controllers compared to progressors have distinct TCR clonotypes that are more ‘broadly reactive,’ similar to the concept of broadly neutralizing antibodies in that they demonstrate cross-reactivity to epitope variants [42,45]. Cross-reactive TCRs have been shown to limit the ability of the virus to escape recognition [46–48], but there is not a strict correlation between T cell clonotypic features and HIV control [49,50]. From a therapeutic standpoint, cross-reactive public TCRs identified from controllers could potentially be adapted for use in adoptive T cell therapies (e.g., TCR-T cells; [51]). While HIV-specific CD8^+^ T-cell responses with cross-reactive TCRs can be elicited after vaccination in HIV-uninfected people [52], it is unclear whether therapeutic vaccines given to people with HIV with preexisting HIV-specific T cell responses can overcome immunodominance patterns to elicit *de novo* responses or even to elicit new clonotypes and alter clonotype hierarchy within preexisting responses [53]. Implications:

- How can broadly-reactive HIV-specific CD8+ T-cell responses be induced therapeutically, and can they contribute to posttreatment control of HIV?
- How can immune therapies induce novel CD8+ T-cell responses during ART and overcome immunodominance of the existing T cell responses?

#### Epitope targeting

Irrespective of the unmodifiable variable of HLA type, CD8^+^ T cells from spontaneous HIV/SIV controllers may preferentially target epitopes that are evolutionarily conserved and that have lower mutational tolerance (reviewed in [25]). Evidence for this phenomenon was first established when it was noted that the magnitude and breadth of HIV-specific CD8^+^ T-cell responses specifically targeting the relatively conserved Gag structural protein but not the highly variable Env surface protein epitopes (or the total HIV proteome) was associated with a lower viral load [13,14,54–56]. At a finer epitope level, specific regions targeted by elite controllers across HLA types infected with clade B virus tend to be more conserved and have lower mutational tolerance (i.e., they are more evolutionarily constrained, or structurally ‘networked’) than those in progressors [57]. Conserved/constrained sequences (mostly derived from the Gag and Pol protein) have been used as therapeutic vaccine immunogens in several approaches, although none has demonstrated clinical efficacy yet [57–62]. It not clear whether T cell-based therapeutic strategies for HIV are more likely to be successful if they target broad versus narrow epitope responses. As noted above, spontaneous controllers appear to target broader responses within conserved regions compared to noncontrollers. Furthermore, epitope breadth elicited by a therapeutic vaccine has been shown to associate with delay to viral rebound in NHPs [63]. On the other end, narrowly targeting CD8^+^ T-cell responses may be favorable to avoid epitope ‘distraction’ from the most conserved regions [64^▪^]. A prophylactic vaccine strategy in NHPs targeting only three CD8^+^ T-cell epitopes was sufficient to maintain viral control after SIV challenge and viral escape from these three epitopes occurred concomitantly with loss of control of viral replication [65]. Similarly, viral escape of a single epitope has also been associated with loss of long-term control in a person with HIV [66]. These data suggest that a few well targeted epitopes might be sufficient to induce viral control. Implications:

- Can therapeutic vaccines elicit CD8^+^ T-cell responses that reliably recognize conserved/constrained regions across individuals with diverse HLA types who are infected with diverse HIV viral strains, and can these T cell responses mediate durable control of HIV *in vivo*?
- Should therapeutic vaccines aim to elicit broad or narrow responses to key epitopes?
- Are the considerations about breadth of epitope targeting different for TCR-based adoptive T-cell therapies?

### II. Quality and localization of HIV-specific CD8^+^ T-cell responses in elite controllers

Independent of T cell specificity and HLA type, HIV-specific CD8^+^ T cells in elite controllers have also been shown to be highly functional, to exhibit a more memory-like and less exhausted differentiation state, and to localize better to sites of reservoir persistence within lymph nodes compared to responses detected in people who do not control HIV. In order to comprehensively evaluate different T cell-based HIV remission strategies, it will be critical to evaluate to what extent they can promote each of these qualities.

#### Enhanced expansion and antiviral functions

Compared to HIV-specific CD8^+^ T cells from noncontrollers (on or off ART), HIV-specific CD8^+^ T cells from elite controllers have increased capacity to produce multiple antiviral cytokines after peptide stimulation *in vitro* (polyfunctionality) and demonstrate increased expansion capacity, increased expression of the effector protein Perforin, and sustained killing of infected target cells over the course of several days of peptide stimulation *in vitro*[67–77]. It is unclear whether this enhanced functional capacity compared to the functionally exhausted cells in noncontrollers is acquired as a consequence of elite controllers likely having a shorter duration and lower cumulative exposure to high viral load prior to viral suppression compared to noncontrollers, or whether this capacity is directly responsible for mediating and maintaining durable viral suppression. Indeed, people or NHPs who experience curtailed viremia due to initiation of ART early in the course of infection also have more highly functional HIV-specific CD8^+^ T cells ([78], and unpublished data, L.T.). Regardless, the functional properties of HIV-specific CD8^+^ T cells in elite controllers serve as a model for the type of CD8^+^ T-cell response that should ideally be elicited in remission strategies. Indeed, recent studies have shown that T cell function and expansion can be enhanced by promoting cellular pathways that are active in HIV-specific CD8^+^ T cells from elite controllers or inhibiting those found in noncontrollers (e.g., via inhibition of co-inhibitory receptor signaling, apoptosis pathways, the mammalian target of rapamycin (mTOR) pathway, and/or overexpression of TCF-1, a Wnt signaling transcription factor that promotes memory T cell-like expansion capacity; [79^▪▪^,^80^–^82^,83^▪^,84^▪^]). Implications:

- How can therapeutic strategies for HIV remission promote the generation of nonexhausted HIV-specific CD8^+^ T cells with functional properties similar to elite controllers (i.e., enhanced expansion capacity and antiviral function)?

#### Tissue localization

To control viral replication, HIV-specific CD8^+^ T cells need to be localized in close proximity to sites of viral reservoir persistence in tissues. HIV persists in lymphoid tissues throughout the body with a high burden of infected CD4^+^ T cells found in the gastrointestinal tract [85,86]. Increased frequencies of functional HIV-specific CD8^+^ T cells have been observed in the rectal mucosa of HIV controllers [87]. Within lymphoid tissue, several studies in humans and NHPs have shown that HIV/SIV preferentially persists in both controllers and noncontrollers in follicular helper CD4^+^ T cells localized within B cell follicles, from which HIV-specific CD8^+^ T cells are mostly excluded [33,88–97]. While it has been suggested that the HIV-specific CD8^+^ T cells located in lymphoid tissue may have impaired cytolytic function [98], these cells (or at least a subset of them) may be poised to respond rapidly to antigen stimulation [99–101]. Moreover, highly functional CXCR5-expressing SIV-specific CD8^+^ T cells are associated with viral control in SIV-infected NHPs [102]. Taken together, these data suggest that while HIV-specific CD8^+^ T cells in the lymph nodes from controllers may have a less effector differentiated phenotype compared to cells found in the blood, they nonetheless possess the capacity to expand and differentiate into potent antiviral effector cells and traffic to B cell follicles where the HIV reservoir persists. Several immune-based remission strategies are being developed to re-direct CD8^+^ T cells to the B cell follicles, including pharmacologic treatment with IL-15 agonists, genetically engineering CXCR5-expressing CD8^+^ T cells for adoptive transfer, and the development of bi-specific antibodies to redirect follicular CD8^+^ T cells to kill infected cells [103,104,105^▪^,^106^,^107^]. Implications:

- How can remission strategies be tailored to optimize the generation of HIV-specific CD8^+^ T cells that migrate to lymphoid tissues/B cell follicles and have the potential to generate a potent effector response ‘at the right place at the right time’?

### III. Timing of CD8^+^ T-cell responses and coordination with other immune responses

While HIV-specific CD8^+^ T cells have been extensively studied in spontaneous controllers during the phase of long-term control, there are very limited data on CD8^+^ T-cell dynamics during acute infection in people destined to become controllers. Specifically, it is unclear whether or how the features of HIV antigen recognition and HIV-specific CD8^+^ T-cell quality and localization discussed in the two previous sections directly contribute to control early in primary infection or whether they arise as a consequence of these individuals achieving greater viral control. Understanding the timing and nature of the CD8^+^ T-cell response in early HIV/SIV infection in spontaneous controllers, how CD8 T cells engage rebounding virus in posttreatment controllers, and how CD8^+^ T-cell responses coordinate with other immune responses to productively engage with and suppress the virus at the time of viral intercept in both settings is therefore crucial to directly inform the development of improved remission strategies.

#### Timing of CD8^+^ T-cell responses

While current data are limited, studying immune responses that occur in acute infection or immediately post-ART in HIV controllers may identify targets for therapeutic intervention to promote HIV remission. In the SIV model, while CD8^+^ T cells generally exhibit a suboptimal ability to suppress SIV in acute infection, in controller macaques, suppressive capacity increases progressively before the establishment of sustained low-level viremia [108]. In humans, a study reported higher frequencies of proliferating CD8^+^ T cells in acute infection in two individuals who maintained low viremia without ART, suggesting that CD8^+^ T cells might play an early role in viral control [109]. Recently, three cases of women identified in early infection who subsequently developed spontaneous control in the absence of ART were described, with two showing robust and one very limited HIV-specific CD8^+^ T-cell responses during acute infection [110,111]. These data suggest that CD8^+^ T-cell responses may have a role early in infection in some spontaneous controllers, but the exact timing of these responses and their impact on control is not yet fully clear. In noncontrollers in the absence of any immunologic intervention, two studies have suggested that HIV/SIV-specific CD8^+^ T cells do not respond early enough after ART discontinuation to prevent viral rebound and only exert an effect on viral load set-point after viral rebound [112,113]. However, the HIV-specific CD8^+^ T-cell response right after ART is stopped has not yet been described in controllers. Implications:

- Studies describing the earliest interactions between the emerging virus and the CD8^+^ T-cell response in both spontaneous and posttreatment controllers will be key to informing the successful development of interventions aimed at priming an immune response to target the early viral intercept.

#### Coordination of immune responses post-antiretroviral therapy

The studies described in the section above suggest that, in the majority of people with HIV who fail to control the virus in the absence of ART, CD8^+^ T cells may respond too slowly to stop viral spread after ART is discontinued. Effective immunity during the early stages of viral spread likely requires other interventions to reduce the size of the HIV reservoir, and/or to augment other immune responses to either directly promote functional CD8^+^ T-cell responses or to contain early viral replication and thus allow CD8^+^ T cells time to expand and mature (reviewed in [114]). For example, type I interferon-producing plasmacytoid dendritic cells (pDCs) provide help to CD8^+^ T cells and have recently been shown to sense the virus in tissues and become activated prior to viral rebound detectable in the blood [115]. Classical dendritic cells that are capable of priming CD8^+^ T cells have additionally been shown to be highly functional in spontaneous controllers [116]. Antibodies have also been suggested as enabling immune complexes promoting stronger CD8^+^ T-cell responses [117]. Understanding the interaction between CD8^+^ T cells and other cell types and immune responses – such as pDCs, other innate immune cells, CD4^+^ T cells, or B cells/antibodies – will likely be required to fully understand this early response during viral rebound post-ART. Implications:

- Combined interventions targeting multiple virologic and immunologic mechanisms will likely be required to achieve HIV remission.
- Interventions that allow for a transient reduction of viral replication post-ART could allow for a better maturation of the CD8^+^ T-cell response in response to rebounding virus and give it enough time to start controlling viral replication.

## CONCLUSION

**FIGURE 2:**
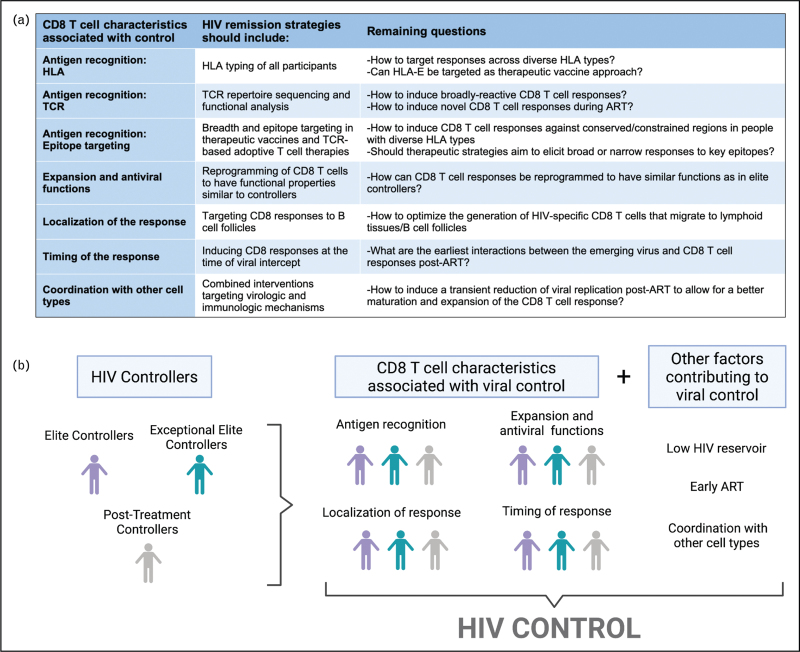
CD8^+^ T-cell characteristics of viral control: Potential implications for HIV remission strategies. (a) List of implication for novel HIV remission strategies and remaining questions to be answered. (b) HIV controllers grouped and analyzed for the different CD8^+^ T-cell characteristics associated with viral control will inform HIV remission strategies and need to be targeted in combination with other factors contributing to viral control.

The past three decades of studies on CD8^+^ T-cell responses in spontaneous HIV/SIV controllers have provided important information on their key role and potential mechanisms contributing to viral suppression. In order to successfully apply this knowledge to T cell-based remission strategies, we suggest the following (Fig. 2a):

(1) CD8^+^ T-cell-based remission strategies should seek to elicit T cell responses with ‘broadly reactive’ TCRs that target highly conserved/evolutionarily constrained regions of the virus, are localized to sites of reservoir persistence, are durable and highly functional, and are capable of responding rapidly (either on their own or in conjunction with other immune responses) to emerging virus after ART is discontinued.
(2) Studies that include HIV remission interventions that target CD8^+^ T cells should ideally report HLA typing of study participants, clinical information about pre-ART viral loads and duration of infection prior to ART initiation, epitope mapping and evaluation for broadly reactive TCRs, analysis of the long-term durability and functional capacity (including expansion capacity and, ideally, viral inhibition), and lymphoid tissue localization or at least homing potential (e.g., CXCR5 expression) of the CD8^+^ T-cell response.
(3) As a field, we need to better understand how the timing of the CD8^+^ T-cell response and its coordination with other immune responses in blood and tissues at the time of viral intercept relates to viral control in natural infection and after therapeutic intervention.
(4) As different CD8^+^ T-cell characteristics associated with viral control are likely shared by the different groups of controllers in natural infection, but may differ by individual, it is also important to recognize that HIV remission strategies might need to be tailored to different groups of individuals depending on the mechanism targeted (Fig. 2b). In addition, combination therapies might be necessary as interventions focusing only on CD8^+^ T cells might not be sufficient to induce viral control [114].

## Acknowledgements

*We would like to thank Julie Mitchell for her help in designing* *Fig. 2* *and Steven Deeks for comments on the text.* *Figs. 1 and 2* *were designed using Biorender. The opinions or assertions contained herein are the private views of the authors, and are not to be construed as official, or as reflecting true views of the National Institutes of Health.*

### Financial support and sponsorship

*This publication was made possible with support from NIH grants UM1AI164560 (R.R., L.T.), R01AI147749 (L.T.), K23AI134327 (R.R.), R01AI170239 (R.R.).*

### Conflicts of interest

*There are no conflicts of interest.*

## REFERENCES AND RECOMMENDED READING

Papers of particular interest, published within the annual period of review, have been highlighted as:

▪ of special interest
▪▪ of outstanding interest
